# Analysis of Short Tandem Repeats by Parallel DNA Threading

**DOI:** 10.1371/journal.pone.0007823

**Published:** 2009-11-13

**Authors:** Pawel Zajac, Christine Öberg, Afshin Ahmadian

**Affiliations:** Department of Gene Technology, School of Biotechnology, Royal Institute of Technology (KTH), Stockholm, Sweden; Niels Bohr Institute and Biological Institutes, Denmark

## Abstract

The majority of studies employing short tandem repeats (STRs) require investigation of several of these genetic markers. As such, we demonstrate the feasibility of the trinucleotide threading (TnT) approach for scalable analysis of STRs. The TnT method represents a parallel amplification alternative that addresses the obstacles associated with multiplex PCR. In this study, analysis of the STR fragments was performed with capillary gel electrophoresis; however, it should be possible to combine our approach with the massive 454 sequencing platform to considerably increase the number of targeted STRs.

## Introduction

Microsatellites, or short tandem repeats (STRs), are abundant 1–6 bp nucleotide motifs repeated in a tandem fashion in genomes from all classes of organisms, ranging from prokaryotes to eukaryotes [1], [2]. Microsatellites are predominantly present within non-coding DNA regions, whereby they affect, for instance, chromatin organization, DNA replication and recombination, as well as gene activity [3]. However, an increased number of repeats has been found in protein-coding portions of the genome, which could influence protein function and thus the phenotype [4]. Characteristics such as high variability and abundance have earned these repeated units widespread usage as genetic markers in mapping and population studies [5]. Additionally, microsatellites have been implicated in numerous diseases. For instance, some cancer types show signs of STR instability [6] and unstable trinucleotide repeats have been linked to neurodegenerative disorders [7].

Different individuals exhibit microsatellite variations, manifested as repeat number differences, hence lending these markers particularly suitable for establishment of human identity within the fields of forensics or paternity testing. For instance, the FBI employs a set of STRs as the core in the Combined DNA Index System (CODIS) to obtain unambiguous identification [8].

In the majority of such investigations, several STRs need to be analyzed. For this reason, parallelized STR assays are necessary. Today, the most widely employed method involves PCR amplification and fragment analysis by gel electrophoresis. It is, however, difficult to increase the multiplexity of PCR as this results in a reaction outcome dominated by unspecific amplicons. Trinucleotide threading (TnT) represents a scalable alternative to conventional PCR amplification circumventing the above-mentioned problem [9]. TnT has successfully been employed to simultaneously amplify 147 DNA regions without generation of spurious products, yielding material suitable for genotyping [10] and expression profiling [11]. In this proof-of-concept study, three markers from the FBI CODIS set were assayed with TnT to evaluate this approach for parallel amplification of STRs.

## Results and Discussion

In this study, the usefulness of the trinucleotide threading (TnT) multiplex amplification strategy for parallel STR analysis was investigated using three markers. As TnT has been shown to specifically amplify desired DNA regions, it could address the inherent limitations of multiplex PCR and, accordingly, enable larger STR sets to be amplified in a parallel fashion. The three markers – TPOX, CSF1PO and D18S51 – were chosen among the ones of the FBI CODIS set and represent tetra-repeats assembled of A, G and T nucleotides. Due to the extensive use of this collection, these STRs are well-defined and scrupulously characterized, proving ideal substrates for this proof-of-concept study.

**Figure 1:**
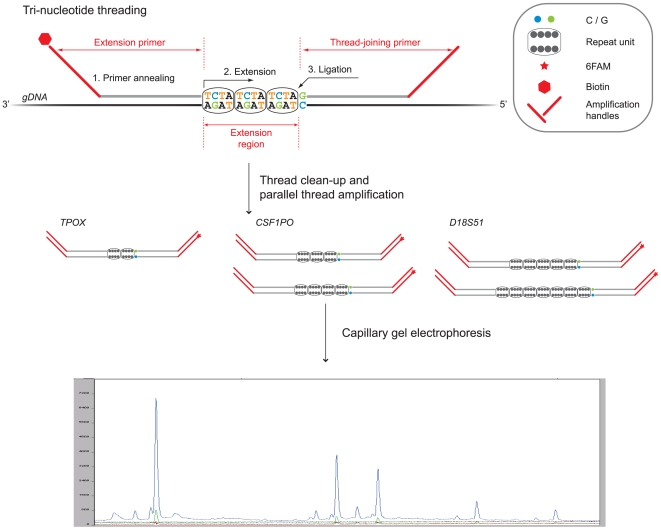
Schematic representation of multiplex amplification of microsatellite regions with trinucleotide threading. Genomic DNA acts as template in the trinucleotide threading reaction, which entails DNA thread formation by a three-step process: 1) annealing of the threading primers; 2) polymerase-mediated closing of the gap between the primers, corresponding to the STR section, with a trinucleotide set; and, 3) ligation of the two thread constituents. A biotin tag on the extension primers allows immobilization of the DNA threads onto streptavidin-coated magnetic beads and thus an efficient clean-up. The DNA threading primers carry universal amplification handles, hence enabling parallel PCR amplification. Finally, product lengths are obtained with fragment analysis using capillary gel electrophoresis.

In the TnT reactions, DNA threads corresponding to the microsatellite regions are created by a three-step process: 1) annealing of a pair of primers designed to flank the repeat regions – an upstream extension primer and a downstream so called thread-joining primer; 2) closing of the gap by employing the trinucleotide set that corresponds to the repeated units; and, 3) ligation of the two fragments (Figure 1). As all complete threads share common universal amplification handles, they can be amplified in a concerted fashion with a single primer pair, one being 6FAM labeled hence allowing for detection after fragment separation using capillary gel electrophoresis. Usage of only one dye implicated some restrictions regarding STR choice to avoid length overlap in the readout step. Naturally, utilization of multiple dyes is an option if overlapping lengths are unavoidable and can also increase the multiplicity of the reaction. However, this strategy necessitates a different generic handle for each extra dye.

**Figure 2:**
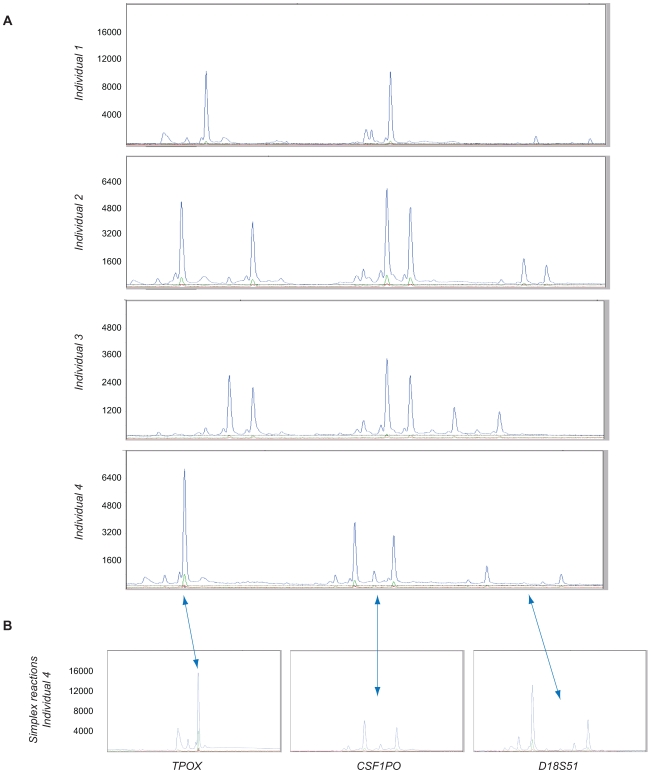
Fragment analysis results. (A) Electropherograms obtained from gel electrophoresis of the multiplex amplification reaction. Data from four individuals are depicted. The relative fluorescence units (RFU) are indicated on the y-axis. The time interval for each of the electropherograms is approximately between 30 minutes (corresponding to fragments length slightly below 130 bp) and 43 minutes (equaling fragment lengths of about 215 bp). Each of the three investigated STRs produces a discrete peak cluster. The differences in the individuals' genotypes for this three-marker set are apparent. (B) Electropherograms derived from the simplex reactions of individual 4, displaying a clear correlation with the STR peak groups from the multiplex assay. RFU are shown on the y-axis. In this example, the TPOX locus is homozygous, whereas the two other loci – CSF1PO and D18S51 – are heterozygous, generating one and two DNA threads, respectively, in the TnT reaction.

The fragment analysis results for the multiplex reactions displayed three distinct peak groups, clearly separated with respect to length, each corresponding to one of the STRs (Figure 2). Analogous peaks were evident in the simplex reactions, allowing for a peak-to-STR correlation (Figure 2). Additionally, the fragment lengths concurred with most frequently encountered repeat numbers in the literature. The signals of D18S51 are weaker than those of the other two STRs, an expected observation given that these fragments are the longest and PCR exhibits a bias towards amplification of shorter fragments. Consequently, trinucleotide threading represents a viable alternative for parallel STR amplification, producing material well suited for gel identification.

The trinucleotide threading assay for analysis of STRs offers two levels of distinction: formation of a DNA thread requires gap bridging with a restricted nucleotide set followed by ligation. This high discriminatory power keeps formation of unspecific products at a minimum, therefore rendering the approach highly specific. In particular, misannealing of the TnT primers predominantly results in extension regions composed of all four nucleotides, hence precluding the action of the polymerase and the ligase. Moreover, the extremely low tendency for spurious DNA thread formation permits cycling of the threading reaction resulting in an initial amplification and an increased specificity. Furthermore, utilization of biotin and magnetic beads, as well as the widely employed 96-well plate format greatly facilitate automation of the procedure minimizing the hands-on time required.

The TnT approach is also compatible with numerous detection systems. Capillary gel electrophoresis was employed in this proof-of-concept study; however, this detection platform is difficult to parallelize. As such, array-based readout with the branch migration assay may prove an alternative [12]. In addition, introduction of 454's massive parallel Pyrosequencing, with an average read-length of 400 bases [13], opens up entirely new possibilities for highly parallel TnT amplification and analysis of STRs. Since a 454 plate can be divided in up to 16 lanes, 50–100 or more STRs can be parallel amplified by the TnT method, and by using multiplex identifiers, several individuals may be analyzed in one lane, thereby reducing the cost. In addition, use of clonal emPCR in the 454 system greatly reduces the issue of length bias in multiplex amplification of STRs. As was shown in this 3-plex amplification, the length variation between threads of different STRs allows the shorter threads to be amplified at the expense of the longer ones in the PCR step. However, the longest threads, corresponding to the D18S51 marker, were still easily detectable with capillary gel electrophoresis. Nevertheless, for larger-scale studies this bias might be aggravated. One strategy to combat this problem could be to divide the desired STRs into pools according to length. This would require running a few parallel TnT reactions, but the potential multiplexity level would most likely be superior to that of conventional PCR. For example, one could envision partitioning a 100 marker set into three groups. These homogenous groups would provide for a more even amplification. Moreover, as the reactions would entail the same reagents, except the TnT primers, a master mix could be prepared thus lessening the added workload. An alternative approach would be to keep all threads in the same reaction tube, but employ different amplification handles depending on thread length. As such, the universal primer amounts could be adjusted to enable a more equal amplification in the PCR step. One drawback would naturally be the necessity to use several generic sequences. Finally, as with emulsion PCR in the 454 scenario, various compartmentalized techniques could be utilized, spatially separating individual thread amplification reactions.

Meticulous STR selection is a crucial step to enable successful TnT amplification. The intrinsic feature of requiring the presence of a trinucleotide gap for thread formation precludes analysis of markers with repeat units including all four nucleotides. However, given the plentitude of STRs in the human genome, finding a suitable set for a particular study should, in most cases, not pose any serious problems. Microsatellites can be chosen among reported repeats, identified by *in silico* sequence mining [14], [15], but also discovered through sequencing of STR enriched regions [16]. Accordingly, the selected markers have to share regular repeat motifs across the targeted populations. Another predicament with the TnT approach pertains to unexpected presence of the fourth type of nucleotide in the repeat region, most frequently due to a mutation, since this prevents the creation of a complete thread. These partial entities will not be amplified in the ensuing universal PCR and, consequently, will not be detected. However, examining the pattern of successful interrogations for a particular STR seeking for anomalies could easily identify such instances. For instance, if a marker produces high signals for all individuals but a few, the latter could harbor the fourth type of nucleotide in the extension region. Nonetheless, with the increased number of STRs that can be analyzed in parallel, failure of one or a small number still generates plentiful genotyping data.

The input material amount requirements represent a significant assay parameter, particularly with regard to samples in limited supply. This scenario is often encountered in crime scene investigations within the forensic field. For other applications, such as paternity establishment or relatedness studies, copious amounts of material can be obtained. In this proof-of-concept study, approximately 10 to 30 ng of genomic DNA (gDNA), corresponding to roughly 1500 to 4500 cells, generated well-defined and easily discernible peaks in the gel electrophoresis readout. However, in a previous study, a TnT rendition for SNP genotyping was shown to produce accurate genotypes starting from 1 ng of gDNA [9]. Accordingly, since both these assays entail samples of equal complexity in the form of the entire genome, the amount of starting material for STR analysis could, most likely, be reduced.

The field of forensic genetics has settled on a small set of STR loci. The pervasive usage of this marker set has led to the development of functional assays based on multiplex PCR and multi-color capillary gel electrophoresis. Accordingly, the TnT approach may not be the method of choice for forensic purposes. However, several other STR studies could benefit from the potentially increased multiplexity of this method.

In summary, trinucleotide threading represents a specific, reliable and convenient multiplex amplification strategy for microsatellites attuned to the most widely employed detection platforms. Hence, applications requiring analysis of numerous STRs could greatly benefit from this new technique.

## Materials and Methods

### STR Selection and TnT Primers

Three markers from the FBI CODIS set – TPOX, CSF1PO and D18S51 – were chosen. These STRs are tetra-repeats with a motif composed of the AGT trinucleotide set (Table 1). For each marker two TnT probes – an extension primer and a thread-joining primer – were designed to flank the STR region (Table 2). Furthermore, care was taken to avoid the presence of the fourth nucleotide (C) within the section enclosed by the probes. Accordingly, this created a gap that could be filled using the ACT trinucleotide set.

**Table 1:** Selected microsatellites.

Locus	Repeat structure	Retrieved sequence/Number of repeats	Chromosomal location	Allele range	Most common allele variants/Frequency
TPOX	GAAT	M68651/11	2p25.3 thyroid peroxidase, 10th intron	4–16	8–12/0.994
CSF1PO	TAGA	U63963 (redirected from X14720)/12	5q33.1 c-fms proto-oncogene, 6th intron	5–16	9–14/0.991
D18S51	AGAA	X91254/21	18q21.33	7–39.2	12–21/0.980

GenBank accession codes for the retrieved sequences are shown. The repeat motif is shown in ISFG (International Society for Forensic Genetics) format. Information about repeat structure, chromosomal location and allele range is derived from [8]. The most commonly encountered variants and the associated frequency calculations are based on Swedish allele frequencies [17].

**Table 2:** Sequences of the trinucleotide threading and parallel amplification primers.

Locus	Extension primer	Thread-joining primer
TPOX	5′-Bio-ggatgatggcggaagttgtcatctcTCCTTGTCAGCGTTTATTTGCCCA-3′	5′-Pho-GTGAGGGTTCCCTAAGTGCCTGTgtcgtgtattccggacagtacgtgg-3′
CSF1PO	5′-Bio-ggatgatggcggaagttgtcatctcTCCTGTGTCAGACCCTGTTCTAAG-3′	5′-Pho-GAAGGCAGTTACTGTTAATATCTTgtcgtgtattccggacagtacgtgg-3′
D18S51	5′-Bio-ggatgatggcggaagttgtcatctcGAGATGTCTTACAATAACAGTTGC-3′	5′-Pho-GAGACAGGTCTCAATTTGTCACTCgtcgtgtattccggacagtacgtgg-3′
Forward amplification primer: 5′ggatgatggcggaagttgtcatctc-3′		
Reverse amplification primer: 5′-6FAM-ccacgtactgtccggaatacacgac-3′		

All sequences are written in the 5′ to 3′ direction. The various modifications are indicated. Lowercase letters relate to the universal amplification handles, whereas uppercase ones denote primer portions specific to STR loci.

To allow parallel amplification of complete DNA threads, generic amplification handles were appended to the 5′-ends of the extension primers and to the 3′-ends of the thread-joining primers. Corresponding amplification primers were designed, the reverse one 6FAM-labeled for detection purposes. As only a single dye was employed, care was taken during the STR selection and primer design to avoid length overlap in fragment analysis. In addition, only the most frequent STR variants were taken into consideration (Table 1).

The 5′-ends of the extension primers carried biotin to facilitate clean up, whereas phosphate groups were added to the 5′-ends of thread-joining primers to enable the TnT reaction. All primers were ordered from MWG-Biotech AG (Ebersberg, Germany).

### Trinucleotide Threading and Parallel PCR Amplification

The trinucleotide threading reaction and the subsequent parallel PCR amplification have been described previously [9], [11]. In this study genomic DNA from nine different individuals was used as template in separate multiplex trinucleotide threading reactions. Additionally, simplex TnT reactions were performed for four of the individuals to confirm the results of the multiplex amplification. Briefly, between 12.5 and 31.5 ng of genomic DNA was combined with 0.01 µM of each extension primer, 0.05 µM of each thread-joining primer, 2 U of Ampligase (Epicentre Biotechnologies, Madison, WI, USA), 0.5 U of Stoffel Fragment of AmpliTaq DNA Polymerase (Applied Biosystems, Foster City, CA, USA) and 0.2 mM of dATP, dCTP and dTTP in 1x Ampligase buffer (20 mM Tris-HCl pH 8.3, 25 mM KCl, 10 mM MgCl_2_, 0.5 mM NAD and 0.01% Triton-X 100; Epicentre Biotechnologies) in a total volume of 10 µl. The reaction was cycled according to the following profile: 1) precycling: 20°C for 5 min and 95°C for 5 min; and, 2) 99 cycles of 95°C for 15 s and 65°C for 12 min allowing for denaturation, primer annealing, extension and ligation. The created DNA threads, each carrying a 5′-biotin, were captured with streptavidin-coated M270 Dynabeads (Invitrogen, Carlsbad, CA, USA) and all remaining constituents of the TnT reaction were removed by consecutive washes with 1x TE (10 mM Tris-HCl pH 7.5, 1 mM EDTA), water and 0.1 M NaOH. Lastly, the immobilized threads were released at 80°C for 1 s in 20 µl water. The clean up protocol was fully automated using a Magnatrix 1200 biomagnetic workstation (NorDiag AB, Hägersten, Sweden).

The purified products were parallel-amplified with a single primer pair, taking advantage of the universal amplification handles present at the ends of all DNA threads. Specifically, the entire volume of the cleaned-up DNA threads (20 µl) was mixed with 0.3 µM of forward primer, 0.2 µM 6FAM-labeled reverse primer, 1 U Platinum Taq DNA Polymerase (Invitrogen), 0.2 mM of all four dNTPs and 5 mM MgCl_2_ in 50 µl 1x Platinum buffer (20 mM Tris-HCl pH 8.4 and 50 mM KCl; Invitrogen). The following temperature protocol was used: 1) polymerase activation: 95°C for 5 min; 2) 35 amplification cycles of 95°C for 30 s, 65°C for 30 s and 72°C for 30 s; and, 3) elongation: 72°C for 2 min.

### Capillary Gel Electrophoresis

The lengths of the fragments were analyzed by capillary gel electrophoresis in an ABI Prism 3700 DNA Analyzer (Applied Biosystems) with the ROX500 ladder (Applied Biosystems) and the POP-6 polymer (Applied Biosystems) according to the manufacturer's instructions. 1.5 µl of the 50 µl PCR reactions were used for the fragment analysis. The injection time was 50 seconds. The results were visualized using the GeneScan 3.7 software (Applied Biosystems).
